# Life Satisfaction, Hopelessness, and Attitudes Toward Irregular Migration Among Young Adults: The Moderating Role of Risk-Taking

**DOI:** 10.3390/bs16101917

**Published:** 2026-10-16

**Authors:** Kamal Adewole Odunjo-Saka, Gabriel Elochukwu Nweke

**Affiliations:** Department of Psychology, Faculty of Humanities, Girne American University, Girne 99138, Cyprus; nwekegabriel@gau.edu.tr

**Keywords:** irregular migration, life satisfaction, risk-taking propensity, hopelessness, youth migration attitudes

## Abstract

Migration is an inevitable aspect of human existence, but youth attitudes toward irregular migration have recently become a growing concern among policymakers, researchers, and government officials in developing countries like Nigeria, where challenging living conditions and lack of opportunities to achieve an individual’s potential often influence migration-related attitudes. While previous studies have largely focused on economic and structural factors, limited research attention has been given to the psychological determinants of irregular migration attitudes. Hence, this study examined life satisfaction and hopelessness as predictors of attitudes toward irregular migration among Nigerian young adults, with risk-taking propensity as a moderator. A cross-sectional survey was conducted among 564 undergraduates from federal, state, and private universities in Nigeria, and four standardized psychological instruments, which include the Satisfaction With Life Scale, Attitudes Toward Unauthorized Migration Scale, General Risk-Taking Propensity Scale (GRiPS), and Hopelessness Scale, were used for data collection. The data collected were analyzed using Partial Least Squares Structural Equation Modelling (PLS-SEM, version 3.0). The results showed that life satisfaction had a significant negative influence on irregular migration attitudes and also indicated that hopelessness had a significant positive influence on attitudes toward irregular migration. Again, risk-taking propensity had a positive influence on migration attitudes and also served as a positive moderator in the relationship between hopelessness and irregular migration attitudes among young adults. Therefore, these findings indicate that hopelessness alone may not be sufficient to explain why some Nigerian young adults develop favourable attitudes toward irregular migration. Rather, the influence of hopelessness appears to depend on the individual’s propensity to engage in or tolerate risk. In conclusion, findings of this study suggest that practical interventions aimed at addressing irregular migration among young Nigerians should extend beyond economic and structural measures to incorporate psychological and behavioural components, particularly programmes that enhance life satisfaction, reduce hopelessness, and promote safer decision-making among risk-prone young adults.

## 1. Introduction

Migration is a complex phenomenon that involves the movement of individuals from one geographical location to another, usually with the intention of settling in a new place (Tsegay, 2023). This movement may occur within national boundaries, which is commonly referred to as internal migration, or across international borders, known as international migration (Bernard & Vidal, 2023). Migration is an ancient activity that is regarded as an inevitable component of human history and civilization, but over time, this movement has assumed different forms, one of which is irregular or undocumented migration. International migration is certainly increasing on a global scale. The most important causes are related to the considerable economic, social, and political imbalances that characterize the gap between relatively rich, democratic, and stable but ageing societies in Europe/America and the much poorer, less stable, but youthful and demographically growing societies in developing countries of the world (Liman et al., 2025). The recent “Japa phenomenon”—a situation where young Nigerians are migrating en masse to industrialized countries—is attributed to socio-economic factors, including unemployment, insecurity, and lack of opportunities (Ayanwale, 2024). The phenomenon of irregular migration has increased significantly in recent times, while the factors driving its growth continue to receive research attention from different perspectives. The World Migration Report (IOM, 2018) attributed the increase in the number of migrants reaching the southern borders of Europe to several structural factors, such as unemployment, political instability, limited access to basic amenities, insecurity, and recurrent geopolitical crises happening in Africa and the Middle East over the past few decades. However, Castelli (2018) identified personal attitudes to emigration, education, religion, and marital status as part of micro-factors that play key roles in individual psychological evaluation regarding migration. Other researchers (Sabti & Ramalu, 2024) claim that the movement of individuals from one geographical location to another is influenced by a myriad of economic, political, and social factors. Several studies also link irregular migration to factors such as unemployment, poverty, and political instability in countries of origin (Department for International Development Großbritannien, 2007; Hennebry et al., 2024; Ameh & Anande, 2025).

Throughout history, people have migrated across regions and national borders for reasons ranging from employment and political stability to environmental sustainability. While migration itself is neither inherently problematic nor abnormal, the increasing prevalence of irregular migration, particularly among young people in developing countries, has become a growing concern for governments, policymakers, and researchers due to its associated humanitarian, social, economic, and security implications. In recent years, Nigeria has witnessed an unprecedented increase in the number of young people expressing strong intentions to migrate abroad, often through irregular and highly dangerous routes. This growing phenomenon, popularly reflected in the “Japa” movement, has drawn considerable public attention and generated extensive debate regarding the underlying factors motivating young Nigerians to seek opportunities outside the country. Although legal migration remains a legitimate route for many individuals, a substantial proportion of prospective migrants have demonstrated a willingness to engage in irregular migration despite the considerable risks involved, including exploitation by human traffickers and smugglers, hazardous journeys across deserts and seas, detention, deportation, physical violence, and even death (Okunade, 2018). Such willingness suggests that migration decisions are not solely based on objective assessments of opportunities abroad but are also influenced by individuals’ subjective evaluations of their present circumstances and prospects. Existing literature has largely explained irregular migration from economic, political, and structural perspectives, emphasizing factors such as unemployment, poverty, economic instability, poor governance, insecurity, corruption, educational limitations, and inadequate social welfare systems as major drivers of migration intentions (Okunade, 2018; Sabti & Ramalu, 2024). While these macro-level explanations have significantly advanced understanding of migration dynamics, they do not fully explain why individuals exposed to similar socioeconomic conditions often differ significantly in their attitudes toward irregular migration. Some young adults remain optimistic and pursue alternative routes for personal advancement within their home country, whereas others develop favourable attitudes toward irregular migration despite being fully aware of its inherent dangers. This variation highlights the importance of examining the psychological processes that influence migration-related attitudes and decision-making.

Existing research has identified psychological distress, including hopelessness, as a potential correlate of favourable attitudes toward irregular migration, but has largely overlooked the conditions under which such vulnerability informs migration-oriented attitudes; in particular, little is known about whether risk-taking propensity strengthens the relationship between hopelessness and favourable attitudes toward irregular migration among young adults. Another research gap has to do with the limited integration of positive and negative dimensions of psychological well-being with behavioural dispositions in explaining migration attitudes. Life satisfaction represents a positive appraisal of one’s current life, whereas hopelessness reflects negative expectations about the future; however, the extent to which these psychological states interact with an individual’s propensity for risk-taking remains underexplored. Consequently, existing research provides insufficient understanding of how psychological well-being and psychosocial vulnerability combine with behavioural tendencies to influence attitudes toward irregular migration, particularly among young adults in a developing country.

Hence, the present study addresses these gaps by developing a moderated psychological framework linking life satisfaction and hopelessness with attitudes toward irregular migration among Nigerian young adults, while examining risk-taking propensity as a moderating variable. The significant contribution of this study lies in demonstrating that hopelessness alone may not adequately explain favourable attitudes toward irregular migration; rather, its influence may depend on the individual’s propensity to engage in or tolerate risk. By incorporating risk-taking into the relationship between psychological vulnerability and migration attitudes, the study moves beyond a simple predictor-based explanation and provides a more novel account of why psychological distress may lead to migration-oriented attitudes for some young adults but not others. This perspective extends psychological explanations of irregular migration and provides a basis for more targeted interventions that address not only socioeconomic constraints but also psychological well-being and risk-related behavioural tendencies.

## 2. Theoretical Review

### 2.1. Theoretical Foundation

The Theory of Planned Behaviour (TPB), proposed by Ajzen in 1985, is a widely used psychological framework for understanding how individuals form the evaluative orientations that guide their behaviour (Ajzen, 1985). The theory posits that human behaviour is not random but planned, and that the most immediate predictor of behaviour is an individual’s evaluative orientation. According to TPB, intentions are determined by three core factors: attitudes toward the behaviour, subjective norms, and perceived behavioural control. These components jointly explain why individuals may develop favourable or unfavourable evaluative orientations toward a particular action, be it risky or illegal (Conner & Armitage, 1998; Conner, 2020). Attitudes toward a behaviour refer to an individual’s overall evaluation of performing a behaviour based on beliefs about its likely outcomes and the value placed on those outcomes. Regarding the issue of irregular migration, young adults who perceive migration as an avenue to improved living conditions may develop positive attitudes toward the act or process of embarking on it, especially when their current life circumstances are perceived as unsatisfactory. Subjective norms involve perceived social pressure from significant others, such as family members, peers, or community figures, regarding whether a behaviour should be performed. When irregular migration is socially endorsed or normalized within peer or social networks, individuals may feel encouraged to view irregular migration more positively and thereby hold favourable attitudes toward it or toward individuals embarking on it. Perceived behavioural control reflects individuals’ beliefs about their ability to perform the behaviour, including access to resources, opportunities, and personal capability. Therefore, this theory suggests that when youths believe that migration is achievable despite its associated risks and challenges, their attitudinal response to irregular migration may increase.

The theory further emphasizes that behavioural intention serves as the closest antecedent of actual behaviour, suggesting that individuals who hold favourable attitudes, perceive social approval, and believe that they have control are more likely to form strong orientations to act. Perceived behavioural control can also influence behaviour, particularly in situations where individuals believe they can overcome the associated barriers. This is relevant to the situation of irregular migration, which regularly involves a high level of uncertainty and limited actual control. Psychological factors such as risk-taking propensity and hopelessness may affect how young adults evaluate migration risks and opportunities, thereby influencing attitudes and perceived control. Individuals experiencing a lower level of life satisfaction may underestimate potential dangers and overestimate the perceived benefits of migration, thereby strengthening their migration-related orientations. Based on the application of the theory of planned behaviour (TPB), the current study offers a theoretically grounded explanation of how psychological and social factors interact to shape youths’ attitudes toward irregular migration, thereby enhancing the understanding of migration-related decision-making within Nigerian society. 

Attitudes toward behaviour, subjective norms, and perceived behavioural control are the three core components of TPB. Hence, the current study does not conceptualize life satisfaction, hopelessness, or risk-taking propensity as substitutes for the three core components of TPB. Rather, TPB provides the theoretical foundation for the outcome variable—attitudes toward irregular migration, while the psychological variables are incorporated as extensions that may explain the formation and strength of such attitudes. Attitudes toward irregular migration directly correspond to the attitude component of TPB, which constitutes the principal TPB construct examined in the present model.

Subjective norms and perceived behavioural control are also core TPB constructs, but they are not directly measured as focal variables in the present study. It should be noted that their omission does not imply that they are theoretically unimportant; rather, the present study narrows its focus to the psychological determinants of attitudes and therefore does not test the complete TPB model. Furthermore, it should be acknowledged that life satisfaction is not a standard TPB construct, but it is incorporated as an additional psychological antecedent of attitude. Life satisfaction reflects an individual’s global evaluation of the quality and circumstances of their life. From an extended-TPB perspective, individuals who are more satisfied with their lives may have less motivation to evaluate irregular migration favourably because their current circumstances provide comparatively greater psychological and contextual fulfilment. Conversely, lower life satisfaction may increase the perceived attractiveness of migration as an alternative route to improved circumstances. Life satisfaction therefore extends TPB by introducing a broader indicator of psychological well-being that may influence the beliefs and evaluations underlying attitudes toward irregular migration. 

Again, hopelessness is not a core TPB construct. It represents a negative psychological state characterized by pessimistic expectations about the future and the perception that desired outcomes are unlikely to be achieved. Theoretically, hopelessness can influence the cognitive evaluation of available behavioural alternatives. Young adults who perceive limited possibilities for improving their future may evaluate irregular migration more favourably if they regard it as a potential means of escaping unsatisfactory circumstances or accessing better opportunities. Incorporating hopelessness therefore extends TPB by recognizing that attitudes toward a behaviour may be influenced not only by conventional behavioural beliefs but also by an individual’s broader psychological appraisal of their prospects.

The inclusion of risk-taking propensity provides a further extension of TPB. However, risk-taking is an individual-difference characteristic rather than a core TPB construct and reflects the extent to which an individual is willing to engage in behaviours involving uncertainty or potentially adverse consequences. This characteristic is particularly relevant to irregular migration because such migration can involve substantial legal, financial, physical, and social risks. However, the present study conceptualizes risk-taking as a moderator rather than a mediator of the relationship between hopelessness and attitudes toward irregular migration. The distinction is theoretically important. A mediator would explain how or through what process hopelessness produces favourable attitudes. In contrast, a moderator explains when or for whom the relationship between hopelessness and attitudes is stronger or weaker. Risk-taking propensity fits the latter role because it is conceptualized as a relatively stable individual disposition that influences how psychological vulnerability is transformed into behavioural evaluations. 

For instance, two young adults may experience comparable levels of hopelessness but differ in their willingness to tolerate uncertainty and potential negative consequences. A highly risk-tolerant individual may be more likely to perceive irregular migration as an acceptable option despite its risks, whereas a risk-averse individual may maintain a negative attitude toward irregular migration even when experiencing substantial hopelessness. Thus, risk-taking propensity is theoretically expected to strengthen or weaken the effect of hopelessness on attitudes, rather than serve as the psychological process through which hopelessness produces those attitudes. Accordingly, the study extends TPB by moving beyond the traditional behavioural-belief determinants of attitudes to incorporate psychological well-being (life satisfaction), future-oriented psychological vulnerability (hopelessness), and an individual-difference characteristic (risk-taking propensity), thereby explaining not only whether young adults hold favourable attitudes toward irregular migration but also why the influence of hopelessness may vary across individuals with different propensities for risk.

Lastly, the present study deliberately focuses on attitudes, because the research question concerns young adults’ evaluative orientation toward irregular migration rather than their immediate intention to engage in it. Moreover, considering the conventional TPB sequence, attitudes, subjective norms, and perceived behavioural control predict behavioural intention, which subsequently predicts behaviour. Therefore, attitudes toward irregular migration should not be interpreted as equivalent to intention to undertake irregular migration. Because an individual may hold a favourable attitude toward irregular migration by viewing it as an understandable or worthwhile means of improving one’s circumstances—without necessarily intending to undertake the journey themselves. Attitudes can therefore capture an important stage of migration orientation before a behavioural intention is formed.

### 2.2. Empirical Review and Hypothesis Development

***Life satisfaction, hopelessness, and attitudes toward irregular migration:*** The increased awareness of international migration has led to a crucial integration of immigration and life satisfaction research in recent studies (Angelini et al., 2015; Kogan et al., 2018; Monteiro & Haan, 2022). Life satisfaction is gaining significant attention in academic and policy debates because of its widely accepted ability to measure both individual and societal well-being (Bonikowska et al., 2014; Helliwell et al., 2018). Some researchers (Georga et al., 2025) described life satisfaction as a reliable dimension of quality of life, encompassing an individual’s physical, mental, and social well-being. Other researchers described life satisfaction as the cognitive dimension of subjective well-being, referring to the level of contentment perceived by an individual and linked to greater involvement in positive functioning and a more positive attitude toward life (Morales Almeida et al., 2024). Consequently, Neumann et al. (2025) reported that adolescents with a migration background had higher levels of life satisfaction than those without a migration background. This finding suggests that a migration background does not necessarily correspond to lower subjective well-being and that other factors, such as family relationships, social support, cultural connectedness, or resilience, may contribute to life satisfaction among adolescents with a migration background. Several studies have investigated life satisfaction as an important psychological factor in the lives and experiences of immigrant populations. Chiang et al. (2021) demonstrate that the feeling of dissatisfaction, whether caused by unemployment or absence of educational choices or insufficient social support, can be a powerful force behind attitudes or the final decision on migration. The migration literature suggests that life satisfaction influences attitudes toward irregular migration through multiple mechanisms: shaping migration aspirations, guiding emotional and cognitive evaluations of risk, altering tolerance and social trust, and mediating the impact of structural constraints. Specifically, low life satisfaction tends to increase the attractiveness and acceptability of irregular migration, making it a critical factor in understanding contemporary migration realities. According to Kobayashi and Tanaka (2025), attitudes toward immigration are influenced by perceptions of risk, economic opportunity, and social narratives, which interact with individual well-being. This implies that individuals who experience dissatisfaction may be more likely to view migration positively, even when it involves legal or physical risks.

A growing body of migration and social psychology literature highlights life satisfaction as a key subjective factor guiding attitudes toward irregular migration, both directly and indirectly (Anczyk & Grzymała-Moszczyńska, 2021; Aslany et al., 2021; Ciorbagiu et al., 2020). Recent research confirms that subjective well-being has become central to migration analysis, supporting traditional economic explanations by capturing how individuals feel about their life circumstances (Răileanu Szeles, 2025). In situations where legal migration opportunities are limited, and individuals are confronted with feelings of dissatisfaction, this dissatisfaction can result in more favourable attitudes toward irregular migration as an alternative route. Another study (Korol & Bevelander, 2023) shows that individuals with higher life satisfaction experience more positive emotions, gratitude, and social trust, which in turn foster more tolerant and less risk-seeking attitudes toward migration-related issues. Whereas individuals with low life satisfaction are more likely to experience frustration, pessimism, and perceived lack of control, conditions which are closely associated with risk acceptance and openness to unconventional or illegal strategies, including irregular migration. 

Furthermore, life satisfaction has been identified as a key psychological factor that shapes risk perception and decision-making. Individuals who are satisfied with their lives tend to adopt a loss-averse orientation, meaning they are less willing to jeopardize their current stability through dangerous migration journeys. In contrast, dissatisfied individuals often perceive that they have little to lose, which lowers the perceived cost of engaging in high-risk migration. This aligns with broader findings that life satisfaction is linked to behavioural outcomes such as agency, mental health, and risk behaviour, especially among young people (Baumert et al., 2025). Thus, low life satisfaction can indirectly normalize irregular migration by reducing sensitivity to its dangers. This suggests that individuals with low life satisfaction may be more likely to develop negative or instrumental attitudes by viewing migration, either legal or irregular, as a competitive or necessary survival strategy rather than a regulated process. These influences are especially dangerous to young Nigerians, where levels of unemployment are alarming, and social unrest is widespread. 

A previous study of relative deprivation and international migration conducted by Stark and Taylor (1989), using relative deprivation theory, found that migration is an effective mechanism for achieving income gains in households that send migrants abroad. This finding suggests that households’ expectation of improved life satisfaction is a key driver of international migration. Individuals may examine and report their life satisfaction differently based on their specific expectations, values, aspirations, and even previous experiences, rather than their material conditions (Diener et al., 1999; Monteiro & Haan, 2022). Because life satisfaction is the cognitive component of well-being, reflecting how an individual perceives their overall life circumstances or feelings about their life, the concept therefore emerged as a core psychological factor influencing youths’ judgement and perception. Low life satisfaction may trigger feelings of frustration, insecurity, and disillusionment, making irregular migration seem like an attractive escape route from unfavourable living conditions. Individuals with lower levels of life satisfaction tend to perceive greater gains from migration and are therefore more likely to express favourable attitudes towards moving. Carling (2002) noted that individuals facing low levels of subjective well-being may express a desire to migrate but may lack the necessary resources or agency to realize that goal. Whereas an individual with a high level of satisfaction tends to promote stability, positive engagement, and resilience, restructuring the evaluative response to irregular or unsafe migration. This implies that a person’s quality of life is positively associated with the life satisfaction of the individual migrant and can influence their attitudes toward migration. 

Hopelessness, defined as a negative outlook toward the future and a perceived lack of control over life outcomes, has emerged as a critical psychological determinant of migration behaviour. Unlike life satisfaction, which reflects present conditions, hopelessness is future-oriented and captures expectations about what lies ahead (Lo-oh, 2021). Life satisfaction and hopelessness are closely related. Low life satisfaction often leads to increased feelings of hopelessness, as individuals perceive limited opportunities for improvement. This relationship is supported by recent findings linking poor living conditions and limited prospects to both dissatisfaction and migration intentions (Laari et al., 2024). Moreover, both life satisfaction and hopelessness influence attitudes toward migration. Individuals with low satisfaction and high hopelessness are more likely to develop favourable attitudes toward irregular migration, as they seek alternatives to improve their circumstances. A recent empirical study on nursing students found that hopelessness significantly predicts migration intentions, with many participants expressing a desire to leave their country due to perceived lack of future opportunities (Berşe et al., 2025), a condition closely related to what many young adults are currently confronting in Nigeria. Similarly, some researchers have earlier reported that individuals with higher levels of hopelessness are more likely to consider migration as a means of escaping adverse conditions. In a recent qualitative study, Kiriscioglu (2025) emphasizes the emotional complexity of migration decisions, in which migrants usually experience a dynamic interaction between hope and despair that governs their willingness to embark on or continue migration journeys. The researcher maintains that hopelessness does not operate in isolation but interacts with aspirations, resilience, and perceived opportunities. Again, some researchers found that hopelessness is closely linked to broader psychological distress, including depression and anxiety, which are themselves associated with migration intentions (Siviş et al., 2024). These findings suggest that migration decisions are embedded in a broader psychological framework, in which emotional states influence both attitudes and behaviours. These findings are consistent with psychological theories linking hopelessness to risk-taking behaviour. Furthermore, a previous study (Çapri et al., 2013) on relations between life satisfaction, burnout, work engagement, and hopelessness of high school students reported a negative relationship between life satisfaction and hopelessness. This means students with high levels of life satisfaction are less likely to experience hopelessness, unlike those with low levels of life satisfaction. Similarly, a longitudinal study by Shek and Li (2016) among adolescents in Hong Kong also found a negative relationship between life satisfaction and hopelessness, suggesting that individuals who perceive their future as bleak may be more willing to engage in uncertain or dangerous actions, including irregular migration. Hopelessness reduces the perceived value of remaining in one’s current environment, thereby increasing the attractiveness of alternative routes—even those associated with significant risks.

**H_1_.**  *Life satisfaction directly and negatively predicts attitudes toward irregular migration among Nigerian young adults.*

**H_2_.**  *Life satisfaction negatively predicts hopelessness among Nigerian young adults.*

**H_3_.**  *Hopelessness positively predicts attitudes toward irregular migration among Nigerian young adults.*

**Risk-taking as moderator:** The concept of risk-taking has important implications for understanding risky behaviour, such as human trafficking, dangerous travel conditions, and a lack of access to basic services and legal protections, which are constantly associated with irregular migration. Previous research on migration aspirations had largely outlined political and economic considerations as major drivers of attitudes toward migration (de Haas, 2003; Van Heelsum, 2017), categorizing them as meso- and macro-level factors. However, researchers have recently become interested in understanding the role of psychological characteristics such as individual traits and preferences in the development of migration attitudes (Feyissa Dori et al., 2024; Hagen-Zanker et al., 2023). An individual’s risk-taking propensity has been identified as a key psychological trait that influences attitudes toward irregular migration (Belloni, 2016; Brigden, 2018; Kiriscioglu, 2025). Risk-taking was described by Williams and Baláž (2014) as both an objective trait and a subjective orientation. The terms attitudes and aspirations are commonly used interchangeably in migration studies, though conceptually different, but psychologically connected as a viable mechanism that shapes the individual migration decision. Recently, Kiriscioglu (2025) reported that aspirations are shaped not only by personal motivations but also by structural conditions that enable or constrain actual migration. The researcher claimed that aspirations toward migration are not merely pre-stages of action but reflections of how migrants understand their present circumstances and anticipate their prospects. The study later found that risk-taking attitudes are important determinants of aspirations to move forward, even after considering the structural and demographic variables, especially in the context of irregular migration, which inherently involves legal, financial, and physical risks. Cross-national research also indicates that individual risk tolerance significantly influences both migration decisions and life satisfaction, which means that individuals with higher risk-taking traits are better able to manage uncertainty and are more likely to pursue migration opportunities, which can, in turn, enhance their life satisfaction through a sense of agency and control (Baláž & Valuš, 2020). Similarly, Huber and Nowotny (2020) reveal that risk-tolerant individuals exhibit a higher willingness to relocate, while risk-averse individuals are less likely to migrate. Another study by Akgüç et al. (2016) further maintains that migrants tend to be less risk-averse than non-migrants, suggesting a self-selection mechanism where individuals predisposed to risk-taking are more likely to engage in migration.

The literature consistently maintains that individuals with higher risk tolerance are more likely to consider migration, particularly under uncertain conditions. A study by Răileanu Szeles (2025) found that low levels of life satisfaction influence only emigration intentions, as internal migration intentions are primarily driven by specific financial and personal characteristics. This suggests that dissatisfaction with life increases migration aspirations, while risk tolerance enables individuals to consider irregular options despite potential consequences. This aligns with the aspiration–capability framework, which suggests that individuals may aspire to migrate but lack the legal or financial means to do so, thereby turning to irregular migration as an alternative. However, Thunström et al. (2025) claimed that the stock of information available before risk-taking is also a determining factor. The researchers maintained that preference for positive over negative information can also influence the decision to take a risk. Similarly, Qaisi and Makhlouf (2025) noted that when individuals make decisions, they systematically use mental shortcuts by taking into consideration partial data, which can be the case with irregular migrants. According to Atkinson (1957), the motivation to take risks is commonly influenced by two factors: the desire to succeed and the fear of failure. He then proposed that motivation to succeed and fear of failure influence risk-taking; that is, the feeling of uncertainty allows some individuals to see risky situations as opportunities to achieve their goals, rather than as potential threats. Another previous study by Jaeger et al. (2007) found that an individual’s attitude towards risk perception has a direct impact on their tendency to embark on irregular cross-border migration. To underscore the crucial role of risk-taking, Townsend and Oomen (2015) emphasize that understanding how migrants perceive risk and what attitude motivates them to undertake unsafe journeys is incomplete without paying detailed attention to both their pre- and post-departure decision-making processes. Despite growing interest in psychological determinants of migration, there remains limited research explicitly integrating life satisfaction, hopelessness, risk-taking propensity, and attitudes toward irregular migration within a single framework. Most studies examine these variables in isolation or focus on regular migration contexts. Hence, we proposed the following research hypotheses:

**H_4_.**  *Risk-taking directly predicts attitudes toward irregular migration among Nigerian young adults.*

**H_5_.**  *Risk-taking significantly moderates the influence of hopelessness on attitudes toward irregular migration among Nigerian young adults.*

### 2.3. Research Model

**Figure 1:**
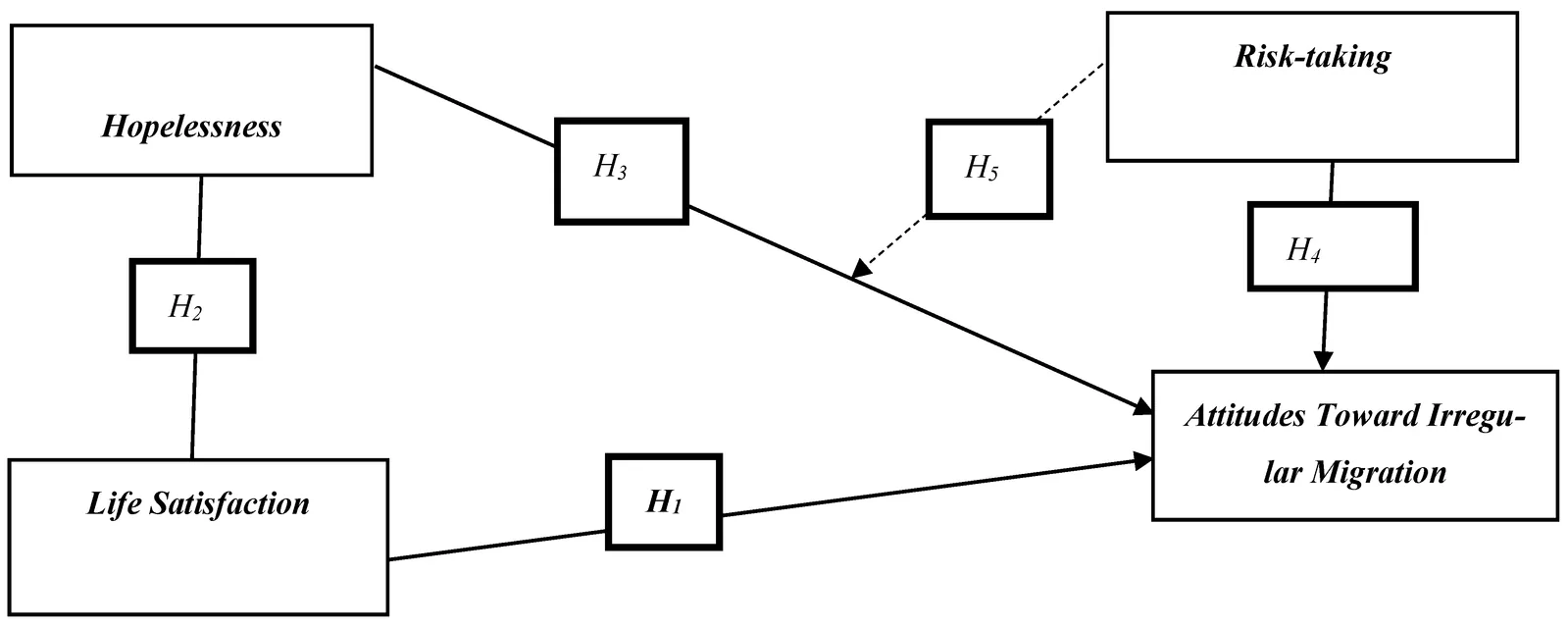
Research model showing the relationships among intervening constructs.

The proposed research model for this study is presented in Figure 1, which provides a visual illustration of the hypothesized relationships among the four latent constructs examined in the study. The model illustrates both the direct and indirect relationships between the study variables. Specifically, it proposes that hopelessness and life satisfaction are directly associated with attitudes toward irregular migration, indicating that individuals’ psychological well-being and perceptions of their future may influence their attitudes toward engaging in or supporting irregular migration. In addition to these direct relationships, the model integrates risk-taking as a potential moderator. Accordingly, risk-taking is proposed to explain, at least in part, how hopelessness and life satisfaction can influence attitudes toward irregular migration. The inclusion of this indirect relationship allows the study to examine not only whether the psychological factors are associated with attitudes toward irregular migration, but also how and through what mechanism these relationships may occur.

## 3. Method and Measures

### 3.1. Research Design

This study is quantitative in nature and therefore employed a descriptive and cross-sectional research approach for data collection to investigate the direct impact of life satisfaction and hopelessness, alongside the moderating effect of risk-taking on attitudes toward irregular migration among Nigerian young adults. The dependent variable is attitudes toward irregular migration, and the independent variables are life satisfaction and hopelessness, with risk-taking as a moderator. The study population comprises all undergraduate students in Nigeria.

### 3.2. Study Area and Participants

This study focused on undergraduate students enrolled in selected Nigerian universities. Nigeria is an example of a developing nation characterized by a teeming youth population, economic instability, rising unemployment, and growing youth interest in international migration. Given these structural and socioeconomic factors, Nigerian undergraduates form a suitable population for studying the psychological determinants of attitudes toward irregular migration among young adults. The study centred on emerging adults in the university setting because their future migration plans depend on the present evaluative orientation to migration-related issues. Full-time undergraduate students from Nigerian universities made up the study population. However, for ease of data collection, participants were drawn from only six universities, which include two federal, two state, and two private universities.

### 3.3. Sampling Procedure

According to the current official website of the National Universities Commission (NUC), Nigeria has a total of 328 universities, comprising 77 federal universities, 69 state universities, and 182 private universities. Geographically, these universities are distributed across the country’s six geopolitical zones. The South-West has the highest number, with 89 universities, comprising 14 federal, 15 state, and 60 private universities. This is followed by the South-East, with 59 universities, comprising 8 federal, 11 state, and 40 private universities. The North-Central zone has 58 universities, comprising 13 federal, 13 state, and 32 private universities, while the South-South has 52 universities, comprising 14 federal, 13 state, and 25 private universities. The North-West has 50 universities, comprising 19 federal, 10 state, and 21 private universities. The North-East has the smallest number, with 20 universities, comprising 9 federal, 7 state, and 4 private universities. This distribution indicates considerable variation in the number and ownership structure of universities across Nigeria’s six geopolitical zones, with the South-West accounting for the largest concentration and the North-East the smallest.

Using purposive, proportionate, and convenient sampling techniques, relevant data were obtained from undergraduate students across the six selected Nigerian universities. A purposive sampling method was used to choose the South-West geopolitical zone out of the six geopolitical zones in Nigeria, due to its unique nature in terms of the highest number of universities, coupled with the highest population of undergraduate students, and its long history of access to relevant information about international migration. A proportionate sampling method was used to distribute the 755 research questionnaires among the six selected universities based on their student population, in which 425 questionnaires were allocated to the two selected federal universities, 227 questionnaires to the two state universities, and 103 questionnaires were shared among the two selected private universities. However, a convenience sampling method was used to administer the research questionnaire to only the available and interested participants, as the researchers lacked the authority to enforce or compel any undergraduate student to participate in the study. Thus, a total of 755 questionnaires were administered with the help of a trained research assistant. Of these 755 questionnaires distributed, only 642 questionnaires were returned, which accounted for an 85% response rate, while 564 were fully completed and considered suitable for coding and data analysis. Respondents were briefed about the objectives of the study before the questionnaires were distributed, and participation was completely voluntary. The study was approved by the ethical committee of the Faculty of Humanities (Number: 2024-25/-HUM037D) with the approval date of 14 March 2025, and carried out in accordance with necessary ethical standards, such as informed consent, confidentiality, and anonymity of participants. For inclusion/exclusion criteria: participants must be registered students enrolled in a full-time academic programme at bachelor’s degree level to be eligible for participation, while students enrolled in part-time, remedial, or distance-learning programmes were excluded from participation.

Sample size: Researchers have noted that G*Power (version 3.1.9.7) does not directly evaluate the sample size for structural equation modelling (SEM). Therefore, this study determined the required sample size based on the existing recommendation by Nevitt and Hancock (2001) that an optimal SEM sample size should be determined using an N:q ratio of at least 10:1, where N represents the number of cases and q stands for the parameters to be estimated. Based on this position, this study with 35 model parameters demands a minimum of 350 participants as an acceptable sample size. Therefore, the 564 participants selected for the study are considered adequate and appropriate for the model.

### 3.4. Measures

A structured questionnaire from reliable and validated tools was used for data gathering. Likert-type rating scales were used to collect responses for all latent variables. The Satisfaction with Life Scale (SWLS), developed by Diener et al. (1985), was used to evaluate life satisfaction. Five items made up the scale, which measures respondents’ overall cognitive assessment of their life situation. A seven-point Likert scale, with 1 representing “strongly disagree” and 7 representing “strongly agree,” was used to rate each item. Attitudes toward irregular migration were measured using Van der Veer et al. (2008) Attitudes Toward Unauthorized Migration Scale. The scale has nineteen items that assessed moral, social, and personal aspects of irregular migration. The General Risk-Taking Propensity Scale (GRiPS) developed by Zhang et al. (2019) was used to assess risk-taking propensity. The scale consists of eight items designed to assess people’s overall inclination to engage in risky behaviours. The items were rated on a five-point Likert scale, from 1 (strongly disagree) to 5 (strongly agree). The 3-item pessimism dimension of the Life Orientation Scale, created by Scheier et al. (1994), was used to measure hopelessness. Negative expectations about the future were measured using the three items of the pessimism dimension of the Life Orientation Scale. A five-point Likert scale, with 1 representing “strongly disagree” and 5 representing “strongly agree,” was used to record responses. The pessimism dimension of the Life Orientation Scale (revised text) was considered appropriate for measuring hopelessness due to its non-clinical nature and short question items that allow for fast administration and promote quick participant response, unlike Beck’s 20-item hopelessness scale, which has been criticized for being too clinical-oriented and for having lengthy question items that can cause participant fatigue, as well as its rigid “Yes or No” response format that may compel participants to either of the two extremes (Pretorius & Padmanabhanunni, 2024). Again, the choice of the pessimism dimension of the Life Orientation Scale is further justified by the previous work of Beck et al. (1974) titled “The measurement of pessimism: The Hopelessness Scale,” where hopelessness is described as pessimism, which clearly lends empirical support for the appropriateness of pessimism in measuring hopelessness. Thus, the adoption of the pessimism dimension of the Life Orientation Scale to measure hopelessness in the current study is empirically justified.

### 3.5. Data Analysis

Descriptive statistics were used to summarize the sample in terms of frequency and percentage, while Pearson’s correlation examined relationships between variables. The proposed moderation model was tested using Partial Least Squares Structural Equation Modelling (PLS-SEM) with SmartPLS 3.0 software, due to its predictive orientation, minimal distributional assumptions, and suitability for complex models involving moderation. Bootstrapping was applied to assess the significance of path coefficients. The measurement model was evaluated for validity and reliability using standard criteria, including outer loadings, Cronbach’s alpha, composite reliability (CR), rho_A, and average variance extracted (AVE), ensuring the constructs were psychometrically sound before testing structural relationships. 

PLS-SEM was adopted for data analysis because the proposed framework involves a moderation effect, requiring the estimation of an interaction between hopelessness and risk-taking propensity. PLS-SEM provides established procedures for estimating latent-variable interaction effects and assessing whether the strength of the relationship between hopelessness and attitudes toward irregular migration varies according to individuals’ levels of risk-taking propensity. This makes the method particularly suitable for testing the conditional relationship proposed in the study. Again, PLS-SEM is appropriate when the analysis involves multiple latent constructs measured through several indicators and when the research objective emphasizes prediction and explanation of endogenous constructs. The method also imposes fewer requirements concerning multivariate distributional assumptions than covariance-based approaches, making it useful where strict normality assumptions may not be tenable. Accordingly, SmartPLS 3.0 was employed to estimate the measurement and structural models and to test the hypothesized direct and moderating relationships. The choice of PLS-SEM was therefore based primarily on the predictive objective and moderated structure of the research model.

## 4. Findings

### 4.1. Demographic Characteristics

Table 1 presents the demographic characteristics of the study participants. A total of five hundred and sixty-four (564) respondents were included in the analysis. With respect to the type of university attended, the majority of participants (58.8%) were drawn from federal universities, followed by those from state universities (27.5%), while a smaller proportion (13.7%) were from private universities. This distribution suggests that students from federal institutions were more represented in the study. In terms of gender composition, the sample was fairly balanced, although males slightly outnumbered females. Specifically, 52.5% of the respondents were males, while 47.5% were females. Regarding age, most of the participants were within the 20–30 years age bracket (60.5%), indicating that the sample largely comprised young adults. Participants below 20 years accounted for 21.6%, while those above 30 years formed the smallest proportion (17.9%).

The marital status of respondents revealed that a large majority were single (84%), with married participants constituting only 9.8%. The respondents who were divorced made up 3.7%, while those classified under other categories accounted for 2.5%. This pattern reflects the predominance of unmarried individuals within the university environment. Concerning religious affiliation, Christians constituted slightly more than half of the sample (50.53%), followed by Muslims (40.43%), while traditional worshippers represented the smallest group (9.04%). This indicates a diverse religious background among the participants, with Christianity being the most dominant. The distribution of respondents across fields of study shows that students from the social sciences formed the largest group (26.4%), followed by Arts and humanities (23.4%) and health sciences (17.6%). Science (12.5%) and engineering (9.6%) had moderate representation, while education (7.8%) and agriculture (2.7%) recorded the lowest proportions. Overall, the sample reflects a wide spread of academic disciplines, with a stronger representation from the social sciences and arts-related fields.

### 4.2. Measurement Model

The measurement model was conducted using Partial Least Squares Structural Equation Modelling (PLS-SEM) to examine the psychometric features of the four latent constructs, which are: Attitudes Toward Irregular Migration, Life Satisfaction, Risk-taking Propensity, and Hopelessness. Assessment of the measurement model is centred on the following statistical indices: Composite Reliability (CR), Cronbach’s Alpha (CA), rho_A, and Average Variance Extracted (AVE)—the convergent validity values of items related to each of the four latent constructs as recommended by Hair et al. (2017). Assessment of the measurement model confirmed that each of the four latent constructs yielded a measurement value above the recommended threshold of 0.70 for CA, *ρ_a_*, and CR. Similarly, AVE values above 0.5 were obtained for each of the four constructs. Generally, AVE is a statistical measure of how well each construct is truly represented by all its indicators and thereby affirms the measure of convergent validity. Therefore, the research model in the present study is appropriate for further statistical analysis. Table 2 presents the details of the psychometric properties of the four latent constructs obtained in this study, such as Cronbach’s Alpha, *ρ_a_*, Composite reliability, Average Variance Extracted (AVE), *R*^2^, and *f*^2^. 

### 4.3. Structural Model Assessment/Hypotheses Testing

A total of five hypotheses were formulated and empirically tested in the present study using the Partial Least Squares Structural Equation Modelling (PLS-SEM) approach. The hypotheses comprised four direct-effect hypotheses and one moderating hypothesis. The four direct hypotheses examined the relationships between the exogenous constructs—life satisfaction, hopelessness, risk-taking, and attitudes toward irregular migration. The only moderating hypothesis examined whether risk-taking significantly influenced the strength and/or direction of the relationships between the relevant predictor variable and attitudes toward irregular migration. Evaluation of the structural model was based on the magnitude and significance of the standardized path coefficients (*β*) and the coefficient of determination (*R*^2^). The path coefficients provided an indication of the direction and strength of the relationships between the constructs, while the *R*^2^ values indicated the proportion of variance in the endogenous construct, attitudes toward irregular migration, explained by the predictor variables in the structural model.

**Figure 2:**
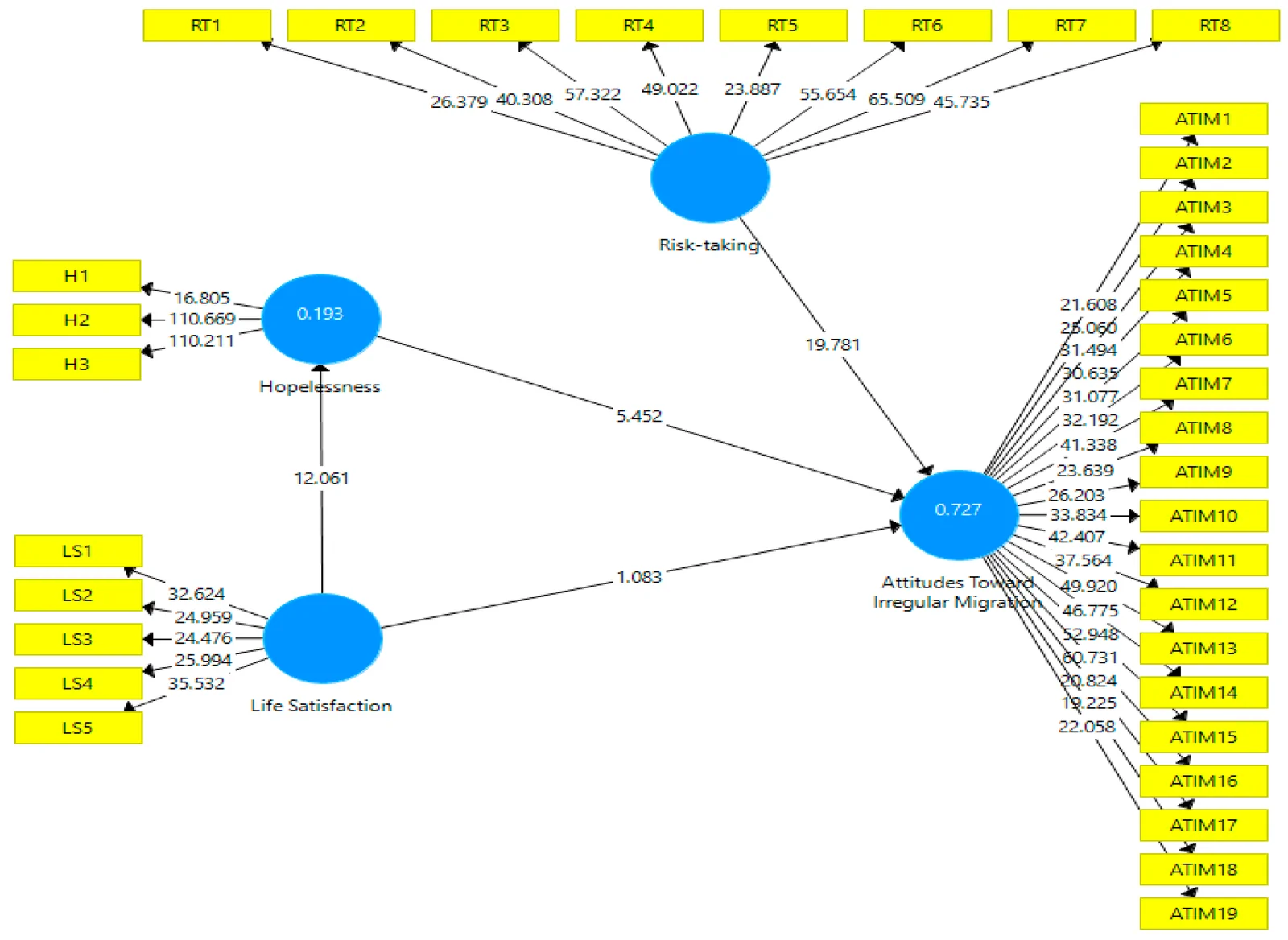
Structural Model with *t*-statistics.

**Figure 3:**
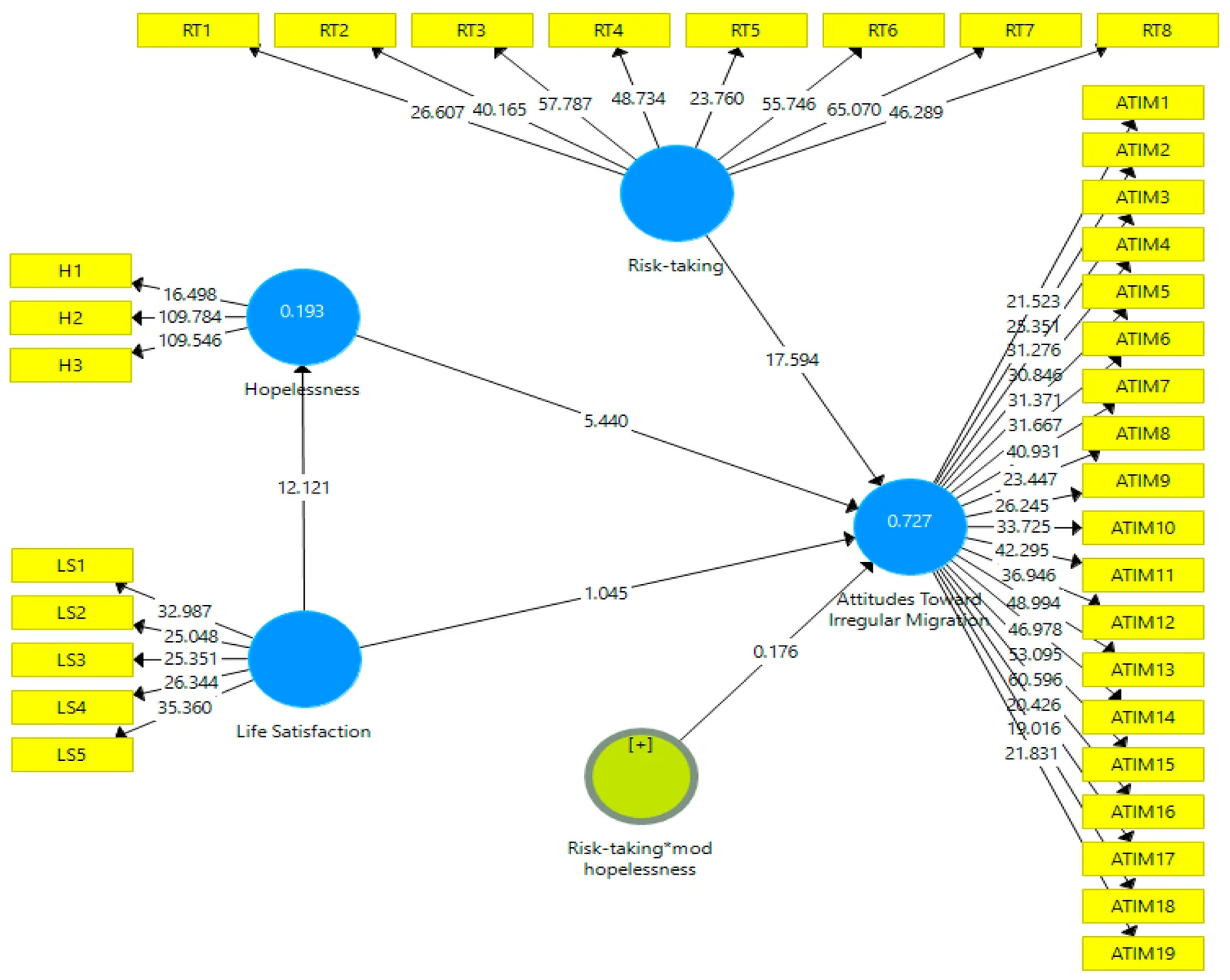
Structural model with risk-taking as a moderator.

Hypothesis testing was conducted using the bootstrapping procedure in SmartPLS. A non-parametric bootstrapping procedure with 5000 bootstrap subsamples was employed to generate standard errors and confidence intervals for the estimated path coefficients. The significance of the hypothesized relationships was determined using the corresponding bootstrapped *t*-statistics and *p*-values. A hypothesis was considered statistically significant where the obtained *p*-value was below the predetermined significance level of 0.05, and the associated confidence interval did not include zero. The use of bootstrapping is appropriate within the PLS-SEM framework because it enables the assessment of the sampling distribution of parameter estimates without requiring the assumption of multivariate normality. The decision to accept or reject each hypothesis was therefore based primarily on the sign and magnitude of the standardized path coefficient (*β*), bootstrapped *t*-statistic, *p*-value, and corresponding confidence interval. The results obtained from the structural model provided empirical evidence for evaluating the proposed direct and moderating relationships and determining the extent to which the hypothesized model was supported by the data. The illustration of path analysis with *t*-statistics is presented in Figure 2, while the moderating role of risk-taking is presented in Figure 3 below.

### 4.4. Moderation Analysis

A moderation analysis was conducted using SmartPLS-SEM with 5000 bootstrap samples to examine whether risk-taking moderates the relationship between hopelessness and attitudes toward irregular migration. For the moderating hypotheses, interaction terms between risk-taking and the respective predictor variables were created and incorporated into the structural model. The significance of the interaction effects was assessed using the bootstrapped path coefficients, *t*-statistics, *p*-values, and confidence intervals. A statistically significant interaction effect was interpreted as evidence that risk-taking significantly moderated the relationship between the corresponding predictor variables and attitudes toward irregular migration. Where significant moderation was established, the nature of the interaction was further examined to determine whether risk-taking strengthened or weakened the relationship. In addition, the effect size (*f*^2^) of the interaction terms was considered to determine the substantive contribution of the moderating effects to the endogenous construct. This approach further enabled the study to establish not only whether risk-taking moderated the hypothesized relationships, but also the direction and practical magnitude of the moderating influences.

## 5. Discussion of Findings

This study investigated how psychological factors affect attitudes toward irregular migration among Nigerian young adults, focusing on life satisfaction, hopelessness, and risk-taking propensity. Five hypotheses were formulated in this study, which comprised four direct hypotheses and one moderating hypothesis. Results of these five hypotheses are presented in Table 3.

### 5.1. Life Satisfaction and Irregular Migration Attitudes

Hypothesis one states that life satisfaction directly and negatively predicts attitudes toward irregular migration among Nigerian young adults. The result shows that life satisfaction had a significant negative influence on irregular migration attitudes (H_1_: *β* = −0.0638, *t* = 3.189, *p* = 0.001). The hypothesis H_1_ is supported, and the result implies that Nigerian young adults with higher levels of life satisfaction are less likely to develop favourable attitudes toward irregular migration compared to their counterparts with lower levels of life satisfaction. The result aligns with a growing body of migration and social psychology literature that highlights life satisfaction as a key subjective factor guiding attitudes toward irregular migration (Anczyk & Grzymała-Moszczyńska, 2021; Aslany et al., 2021; Ciorbagiu et al., 2020). A recent study by Korol and Bevelander (2023) also reported that individuals with higher life satisfaction experience more positive emotions, gratitude, and social trust, which in turn foster more tolerant and less risk-seeking attitudes, while individuals with low life satisfaction are more likely to experience frustration, pessimism, and perceived lack of control, conditions which are closely associated with risk acceptance and openness to unconventional or illegal strategies, including favourable attitudes toward irregular migration. 

The implication of this finding suggests that where legal migration opportunities are limited, and individuals are confronted with feelings of dissatisfaction, such dissatisfaction can result in more favourable attitudes toward irregular migration as an alternative route. Life satisfaction has emerged as an important factor influencing youths’ judgments, aspirations, and perceptions of their future. Low levels of life satisfaction may generate feelings of frustration, insecurity, and disillusionment, thereby making irregular migration appear as an attractive escape from unfavourable living conditions. This means that the perceived quality of life of individuals is closely associated with their life satisfaction and, in turn, influences their attitudes toward migration-related behaviour (Răileanu Szeles, 2025). For instance, individuals with higher levels of life satisfaction are more likely to demonstrate stability, positive engagement with their environment, and resilience, which may reduce their inclination toward irregular or unsafe migration. However, this finding contrasts with the results of Bhattarai et al. (2025), who, in a study of ageing attitudes among older adults with disabilities, reported that positive attitudes toward ageing were associated with higher levels of life satisfaction. While both studies highlight a relationship between life satisfaction and attitudes, the direction of the relationship differs across the two contexts. This difference may be attributable to the distinct nature of the attitudes examined. Theoretically, this finding offers additional insight into the psychological understanding of how life satisfaction can influence irregular migration attitudes among young adults, particularly from a developing country like Nigeria. 

Hypothesis H2 states that life satisfaction negatively predicts hopelessness among Nigerian young adults. The result reveals a significant negative influence in the relationship (H_2_: *β* = −0.44, *t* = 12.144, *p* = 0.001), which suggests that life satisfaction reduces the feeling of hopelessness among Nigerian young adults. Therefore, H_2_ is supported. The results align with the findings of previous studies. For instance, a study conducted by Çapri et al. (2013) on the relationship between life satisfaction, burnout, work engagement, and hopelessness of high school students reported a negative relationship between life satisfaction and hopelessness. The study maintains that students with high levels of life satisfaction are not likely to experience feelings of hopelessness, unlike their counterparts with low levels of life satisfaction. Another longitudinal study conducted by Shek and Li (2016) among adolescents in Hong Kong also found a negative relationship between life satisfaction and hopelessness. Individuals who perceive their future as bleak may be more willing to engage in uncertain or dangerous actions, including irregular migration. Practical implications of the findings suggest that young adults who perceive their lives as unsatisfactory may develop heightened risk-taking tendencies, leading them to underrate the dangers associated with irregular migration while overestimating its potential benefits. In the same vein, low life satisfaction may foster feelings of hopelessness, which can weaken future-oriented thinking and increase openness to hazardous and harmful decisions, including the risks associated with the irregular migration option as a perceived alternative to stagnation in the home country.

### 5.2. Hopelessness and Irregular Migration Attitudes

Hypothesis three states that hopelessness positively predicts attitudes toward irregular migration among Nigerian young adults. The result confirms a significant positive influence in the relationship (H_3_: *β* = 0.168, *t* = 5.592, *p* = 0.001), which reflects hopelessness as a determinant of attitudes toward irregular migration. Therefore, H3 is supported. This result aligns with the findings of other studies. For example, Laari et al. (2024) reported a link between poor living conditions and limited prospects to both dissatisfaction and migration intentions. This implies that individuals with low satisfaction and high hopelessness are more likely to develop favourable attitudes toward irregular migration, as they seek alternative means to improve their circumstances. Cheon (2025), in examining end-of-life care, maintained that positive attitudes toward end-of-life care and higher levels of competence can operate as personal resources that enhance professional meaning, strengthen a sense of efficacy, and support purpose and fulfilment in professional practice. Although the context of end-of-life care differs from that of irregular migration, this perspective provides further support for conceptualizing attitudes as important personal resources that may shape how individuals understand and respond to complex and challenging social issues. In the context of the present study, attitudes toward irregular migration may similarly influence individuals’ willingness to engage with, understand, and respond to the experiences and needs of irregular migrants. Another recent study conducted by Berşe et al. (2025) among nursing students found that hopelessness significantly predicted migration intentions, with many participants expressing a desire to leave their country due to perceived lack of future opportunities. Even though young people are usually aware of the dangers of irregular migration, such as violence, exploitation, or death, hopelessness reduces the weight of these risks in decision-making, and despite knowledge of severe dangers along migration routes, many still undertake these journeys, indicating that desperation can override rational risk assessment. Thereby, making them hold the wrong orientation that hopelessness produces a “nothing to lose” mentality. This means that youths’ perception of their lives as unsatisfactory may prompt them to develop heightened risk-taking tendencies, which may encourage them to underrate the dangers associated with irregular migration while overestimating its perceived potential benefits, thereby suggesting that youths who are preoccupied with fear of the future are most vulnerable to embark on irregular migration. 

### 5.3. Risk-Taking and Irregular Migration Attitudes

Hypothesis four states that risk-taking propensity positively predicts attitudes toward irregular migration among Nigerian young adults. The result confirms a significant positive influence in the relationship (H_4_: *β* = 0.711, *t* = 20.865, *p* = 0.001), which suggests that risk-taking propensity encourages the development of irregular migration attitudes, thereby serving as an important determinant. This result supports hypothesis four; therefore, H4 is supported. This finding aligns with existing findings from other researchers. For instance, Kiriscioglu (2025) reported that subjective risk-taking attitudes strongly predict willingness to pursue onward migration, even under uncertain and constrained conditions. This implies that young people who see themselves as “risk-takers” are more likely to hold favourable attitudes and view irregular migration as a viable option. Again, risk-taking serves as a mechanism that bridges the gap between desire and action, in which young people who are more willing to take risks may likely consider or perceive irregular migration routes (e.g., crossing deserts or seas) as acceptable strategies for achieving their goals, unlike those with low risk tolerance who may choose to remain in place, even if dissatisfied, because staying is perceived as a “safer” option. Again, Jaeger et al. (2007) found that an individual’s attitude towards risk perception has a direct impact on their tendency to embark on irregular cross-border migration. Furthermore, to underscore the crucial role of risk-taking, Townsend and Oomen (2015) claim that understanding how migrants perceive risk and what attitude motivates them to undertake unsafe journeys is incomplete without paying detailed attention to both their pre- and post-departure decision-making processes. Therefore, the result of this hypothesis highlights risk-taking as an important psychological determinant of youth irregular migration attitudes.

### 5.4. Moderating Role of Risk-Taking

Hypothesis five states that risk-taking moderates the influence of hopelessness on attitudes toward irregular migration among Nigerian young adults. This result shows a significant moderating effect of risk-taking in the relationship (H_5_: *β* = 0.054, *t* = 2.157, *p* = 0.031). This result supports hypothesis H5. The positive and statistically significant interaction coefficient suggests that higher levels of risk-taking strengthen the relationship between hopelessness and favourable attitudes toward irregular migration. However, both the moderating effect and effect size of risk-taking are relatively small (*β* = 0.004, *f*^2^ = 0.666), meaning that the impact of risk-taking is not strong on the relationship. Nevertheless, this finding implies that hopelessness alone may not be sufficient to explain why some Nigerian young adults develop favourable attitudes toward irregular migration. Rather, the effect of hopelessness appears to depend on the individual’s propensity to engage in or tolerate risk. Young adults experiencing high levels of hopelessness who also possess a high tendency toward risk-taking are more likely to perceive irregular migration as an acceptable or attractive option despite its potential legal, financial, physical, and psychological consequences. In contrast, individuals with similar levels of hopelessness but lower risk-taking tendencies may be less inclined to develop favourable attitudes toward irregular migration because they may attach greater importance to the potential dangers and uncertainties associated with irregular migration.

The significant moderating effect can therefore be interpreted to mean that risk-taking acts as an amplifying factor in the relationship between hopelessness and attitudes toward irregular migration. When risk-taking is high, the positive association between hopelessness and favourable attitudes toward irregular migration becomes stronger. This may be explained by the fact that hopeless individuals often perceive limited opportunities for achieving desired outcomes through conventional means. When such individuals also have a greater willingness to accept uncertainty and potential negative consequences, irregular migration may become more psychologically acceptable as an alternative strategy to improving their perceived life circumstances.

## 6. Conclusions and Recommendations

### 6.1. Conclusions

This study examined the direct influence of life satisfaction and hopelessness, with the moderating effect of risk-taking, on attitudes toward irregular migration among Nigerian young adults. Findings of this study pointed out that emerging youths who perceive their lives as unsatisfactory are most likely to develop heightened risk-taking tendencies, which can lead them to underrate the dangers associated with irregular migration while overestimating its potential benefits. Again, low life satisfaction may foster feelings of hopelessness, which can weaken future-oriented thinking and increase openness to hazardous and harmful decisions, including the risks associated with the irregular migration option as a perceived alternative to stagnation in the home country. Similarly, heightened hopelessness may increase the perceived acceptability of irregular migration as a coping strategy by young adults in developing countries. The results further show that risk-taking serves as a moderator that strengthens the influence of hopelessness on attitudes toward irregular migration. By investigating the influence of hopelessness and risk-taking on the relationship between life satisfaction and attitudes toward irregular migration among Nigerian young adults, the study offers a rare quantitative understanding of how psychological factors can influence youths’ perception of irregular migration and therefore contributes to the recent calls for incorporating a psychological perspective into migration studies. Overall, the findings support a multidimensional framework in which migration attitudes are formed by the interaction of psychological, social, and structural factors. Understanding these dynamics is essential for developing effective policies and interventions aimed at addressing the root causes of irregular migration.

### 6.2. Recommendations

Based on the findings of the study, the following recommendations are suggested to address psychological factors associated with favourable attitudes toward irregular migration among Nigerian young adults. First, the significant negative relationship between life satisfaction and attitudes toward irregular migration suggests the importance of strengthening young people’s overall psychological well-being and satisfaction with their present circumstances. Universities, government agencies, and youth-focused organizations should therefore develop programmes that promote psychological well-being, social connectedness, career development, and meaningful engagement. Such initiatives should not be designed solely as anti-migration campaigns but should aim to improve the conditions and opportunities that contribute to young adults’ perceptions of a worthwhile and fulfilling life. Second, the positive association between hopelessness and favourable attitudes toward irregular migration further suggests the need to incorporate future-oriented psychosocial support into interventions targeting migration-related vulnerability. Universities and youth-serving institutions should strengthen counselling and psychological-support services that help young adults develop realistic and constructive expectations about their future. Early identification of young adults experiencing persistent hopelessness may also allow appropriate support to be provided before negative expectations become associated with favourable evaluations of risky migration assessment. Importantly, such interventions should complement rather than replace efforts to address the socioeconomic conditions contributing to young people’s concerns about their future. Third, the positive relationship between risk-taking propensity and attitudes toward irregular migration highlights the need for risk-awareness and safer decision-making interventions. Migration-information programmes should explicitly address the potential legal, financial, physical, and social risks associated with irregular migration and should be designed in ways that engage young people who demonstrate greater willingness to tolerate risk. Universities and youth organizations could incorporate decision-making, risk-assessment, and critical-evaluation skills into student development and career programmes. Finally, the findings suggest that policies addressing irregular migration among Nigerian youth should adopt a multidimensional approach that combines socioeconomic, psychological, and behavioural considerations. In particular, migration interventions could integrate measures aimed at improving life satisfaction, reducing hopelessness, and strengthening informed risk assessment.

### 6.3. Limitations and Future Research

Despite the contributions of the present study to the growing body of research on migration studies, certain limitations should be acknowledged. First, the sample was drawn only from Nigerian university students, which may limit the generalizability of the findings to other youth populations, such as out-of-school youths or young adults already engaged in migration processes. Future studies may extend this research to other groups or settings to improve the external validity of the findings. In addition, the study relied on self-reported measures, which may be affected by social desirability or response bias, especially given the sensitive nature of irregular migration. Future research could combine survey data with qualitative methods such as interviews or focus group discussions to gain deeper insights into youths’ migration attitudes and motivations. Furthermore, the study examined risk-taking propensity as a moderating variable. Future studies could explore this variable as a mediator or examine other psychological factors, such as psychological resilience, perceived economic opportunities, family pressure, peer influence, and emotional intelligence, to broaden the psychological understanding of the migration decision-making process among youths. Another important limitation emerged from the nature of measuring hopelessness. Hopelessness was assessed in the present study using the three-item pessimism dimension of the Life Orientation Test, which provides an indirect measure of negative future expectations. Although this approach captures the general aspect of hopelessness, it does not provide a comprehensive assessment of the construct. Future research should therefore consider using a more established and comprehensive measure, such as Beck’s Hopelessness Scale (BHS)**,** to assess hopelessness directly and facilitate greater comparability across studies. Lastly, the cross-sectional design of the study limits causal interpretation of the relationships among variables. Future research may adopt a longitudinal design to observe how changes in life satisfaction, psychological states, and migration attitudes evolve, thereby providing stronger evidence for policy and intervention strategies.

## Figures and Tables

**Table 1:** Demographic characteristics of participants (*n* = 564).

Variables		N	%
**Type of University**	Federal University	332	58.8
	State University	155	27.5
	Private University	77	13.7
**Gender**	Males	296	52.5
	Females	268	47.5
**Age Group**	Below 20 years	122	21.6
	20–30 years	341	60.5
	Above 30 years	101	17.9
**Marital Status**	Single	474	84
	Married	55	9.8
	Divorced	21	3.7
	Others	14	2.5
**Religion**	Muslims	228	40.43
	Christians	285	50.53
	Traditional Worshippers	51	9.04
**Field of Study**	Science	71	12.5
	Engineering	54	9.6
	Health Sciences	99	17.6
	Arts and Humanity	132	23.4
	Social Sciences	149	26.4
	Agriculture	15	2.7
	Education	44	7.8

**Table 2:** Construct Reliability and Validity Values.

Construct	Cronbach’s Alpha	*ρ_a_*	Composite Reliability	AVE Values	Effect Size	
					*R* ^2^	*f* ^2^
Attitudes Toward Irregular Migration	0.883	0.85	0.814	0.515	0.727	-
Hopelessness	0.788	0.881	0.873	0.701	0.193	0.066
Life Satisfaction	0.712	0.821	0.687	0.537	-	0.24
Risk-taking	0.903	0.918	0.92	0.593	-	0.666

**Table 3:** Hypotheses testing with statistical values (*t*-test and *β* values).

Hypotheses	Original Sample (*O*) (Beta Value)	Sample Mean (*M*)	Standard Deviation (STDEV)	*t* Statistics (|*O*/STDEV|)	*p* Values	Remark
**Paths Analyses—Direct Effects**						
Life Satisfaction → Attitudes Toward Irregular Migration	−0.0638	−0.0642	0.02	3.189	0.001	Supported
Life Satisfaction → Hopelessness	−0.44	−0.447	0.064	12.144	0.001	Supported
Hopelessness → Attitudes Toward Irregular Migration	0.168	0.169	0.03	5.592	0.001	Supported
Risk-Taking → Attitudes Toward Irregular Migration	0.711	0.711	0.034	20.865	0.001	Supported
**Moderating Roles—Indirect Effect**						
Risk-Taking*mod Hopelessness → Attitudes Toward Irregular Migration	0.054	0.048	0.025	2.157	0.031	Supported

## Data Availability

The data presented in this study are available upon request from the corresponding author. The data are not publicly available due to privacy or ethical restrictions.
